# Primary headaches increase the risk of dementias: An 8-year nationwide cohort study

**DOI:** 10.1371/journal.pone.0273220

**Published:** 2022-08-18

**Authors:** Seon-Jip Kim, Sang Min Park, Hyun-Jae Cho, Ji Woon Park

**Affiliations:** 1 Department of Preventive Dentistry and Public Oral Health, School of Dentistry, Seoul National University, Seoul, Republic of Korea; 2 Dental Research Institute, Seoul National University, Seoul, Republic of Korea; 3 Department of Family Medicine, Seoul National University Hospital, Seoul, Republic of Korea; 4 Department of Biomedical Sciences, Seoul National University Graduate School, Seoul, Republic of Korea; 5 Department of Oral Medicine and Oral Diagnosis, School of Dentistry, Seoul National University, Seoul, Republic of Korea; 6 Department of Oral Medicine, Seoul National University Dental Hospital, Seoul, Republic of Korea; Ehime University Graduate School of Medicine, JAPAN

## Abstract

**Background:**

Headache, a highly prevalent neurological disorder, has consistently been linked with an elevated risk of dementia. However, most studies are focused on the relationship with migraine in limited age groups. Therefore, the objective of this research was to look at the link between various type of headaches and dementias based on longitudinal population-based data.

**Methods and results:**

Participants diagnosed with headache from 2002 to 2005 were selected and major covariates were collected. The diagnoses of Alzheimer’s disease, vascular dementia, and other dementias were observed from 2006 until 2013. The adjusted hazard ratios (aHRs) and 95% confidence intervals (CIs) of dementias according to headache type were calculated by Cox proportional hazards regression. A number of 470,652 participants were observed for a mean of 7.6 years (standard deviation: 1.2), for approximately 3.6 million person-years. Both tension type headache (TTH) and migraine elevated the risk of all-cause dementias (TTH, aHR 1.18, 95% CI 1.13–2.24; migraine, aHR 1.18, 95% CI 1.13–2.24). Headaches had a greater influence in females and non-smokers as a risk factor of dementias. Patients with migraine who consumed alcohol had a higher risk of dementia, however this was not true with TTH patients. Among participants without comorbidities, TTH patients were more susceptible to dementia than migraine patients. Headache patients had a higher proportion of females regardless of headache type and approximately 1.5 times more individuals had three or more comorbidities compared to those without headache.

**Conclusions:**

Headache could be an independent predictor for subsequent dementia risk. Future studies should focus on clarifying pathogenic pathways and possible dementia-related preventive measures in headache populations.

## Introduction

Headache and dementia are both prevalent neurological diseases and are well known causes of daily dysfunction and lowered quality of life [1]. Dementia is the most common neurological disorder of the elderly with a recent study showing a prevalence of 6.0% for dementia, 3.9% for Alzheimer’s disease (AD), 1.6% for vascular dementia (VD), and 0.5% for other dementias [2]. Due to its progressive nature, dementia is best characterized as a syndrome accompanied by memory loss, impaired executive function, and behavioral disinhibition [3]. Public health burden is estimated to grow rapidly as the number of dementia patients is projected to double fold every 20 years to reach 65.7 million in 2030 as the society ages [4]. Headache is an even more common disorder affecting as much as 46% of the adult population ranking it as the fifth highest cause of disability [1]. Although the prevalence of headache shows a marked decrease in adults >50 years it still remains a common complaint in the elderly [5, 6]. Tension-type headache (TTH), the most common primary headache, generally presents as bilateral, non-throbbing mild to moderate pain. Life time prevalence is as high as 78% marking it as a significant health cost in spite of its lower pain intensity compared to migraine [7]. Migraine is a complex headache condition presenting as recurrent attacks of moderate to severe pulsating unilateral head pain accompanied by nausea and/or photophobia and phonophobia [8]. Its prevalence is approximately 16% for those aged 45–64 years and decreases with age [9]. Associated structural brain changes such as grey matter reduction, white matter hyperintensities on magnetic resonance imaging, and brain parenchymal defects have been identified in migraine patients [10].

Due to the limited efficacy of dementia treatment, research has focused on the identification and prevention of its risk factors [11]. As a result of such efforts several epidemiologic studies have established a close relationship between headache and dementia [12, 13]. Although the exact pathophysiological mechanism that links headache and dementia is yet to be fully elucidated, both disorders share common risk factors and comorbidities including vascular and brain abnormalities, inflammation, cardiovascular disease, and depression [14–17]. Unfortunately, the literature until now still provides contradictory results regarding the type of headache and dementia that are specifically interrelated [13, 18, 19]. Also the majority of research is focused on migraineous headache despite of the superior prevalence of TTH and its known association with cognitive decline [20]. The fact that the majority of related studies are based on small sample sizes of a restricted age range and lack explicit time-related separation of headache and dementia is another limiting factor in defining a temporal relationship between headache and dementia.

Therefore, this longitudinal population-based cohort study based on large datasets applying advanced analytic strategies aimed to explore the possible causal relationship between different types of headaches and dementias to define the role of headache treatment for the prevention of incident dementia.

## Methods

### Population

The National Health Insurance Service-National Health Screening Cohort (NHIS-HEALS) data were used in this retrospective cohort study. NHIS-HEALS followed 514,866 (approximately 10% of the population) Korean participants over 40 years of age who received comprehensive health screenings starting from 2002 and2003until December 2013 for 11 years. Since 1989, the NHIS has conducted health screening every two years for all citizens over the age of 40. As a result, approximately 98% of the Korean population is enrolled for NHIS, and the data obtained from the health screenings are provided to researchers through a random sampling process [21]. NHIS-HEALS data consists of qualification data, health screening data, and claims data. Qualification data contains demographic characteristics and causes and dates of death. Health screening data includes questionnaires on lifestyle such as smoking status and drinking frequency, as well as clinical laboratory results including blood pressure, fasting glucose, and lipid panel. Claims data is based on diagnoses done by a clinician and is divided into main and secondary diagnoses, which can be analyzed by combining it with the qualification and health screening data. Various epidemiological studies have used this database to report associations, and its validity has been described in detail in other research papers [22–24]. The Institutional Review Board (IRB) of School of Dentistry, Seoul National University approved this study (S-D20220003). Written consent of the participants was not received since NHIS clinical data is thoroughly anonymized in accordance with the guidelines of the Personal Information Protection Act.

**Fig 1:**
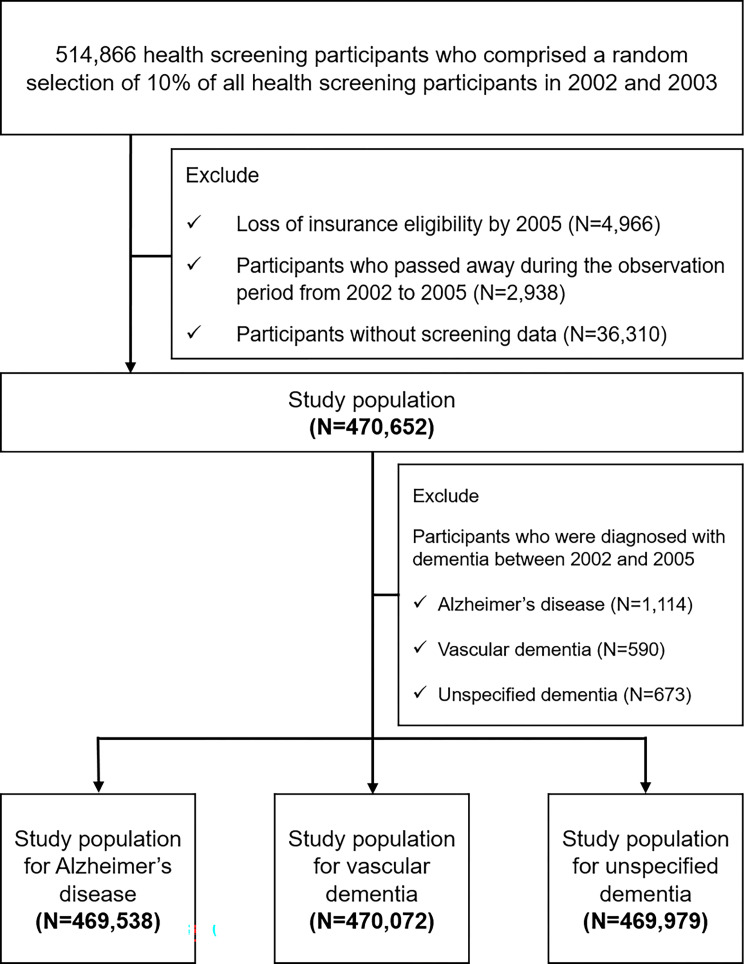
Flowchart for study population selection.

Of the 509,900 participants who maintained insurance eligibility in 2005 (index date: January 1, 2006), 2,938 deaths occurred before the index date and 36,310 participants without screening data were excluded. Then, cases of dementia diagnosed from 2002 to 2005 were also excluded as shown in Fig 1.

### Covariates

Demographic and social characteristics (age, sex, and household income), lifestyle factors (smoking status, alcohol intake, and physical exercise), clinical information (blood pressure, fasting serum glucose, total cholesterol, and body mass index), and comorbid conditions (Charlson Comorbidity Index, CCI) [25] of the participants were obtained from health insurance eligibility and national health screening of NHIS-HEALS, respectively. History of dementia-related comorbidities such as depression (F32, F33), sleep disorder (G25.8, G47, and F51), Parkinson’s disease (G20), and head injury (S02.0, S02.1 S02.3, S02.7, S02.8, S02.9, S07.1, S09.1, S06, and S09.7) were collected for participants diagnosed with a headache. CCI was computed by weighting and rating for comorbid conditions to reflect total morbidity. Age was treated as a continuous variable while all other factors were treated as categorical variables.

### Outcomes

Headache (TTH, migraine, and other primary headaches) as the main exposure and dementia (AD, VD, and unspecified dementia) as the main outcome were identified based on the International Classification of Diseases, 10th Edition (ICD-10) diagnoses [26]. The ICD-10 codes according to the type of headache are as follows; TTH (G44.2), migraine (G43), other headache syndromes (G44.0 cluster headache syndrome, G44.1 vascular headache, G44.3 chronic post-traumatic headache, G44.8 other specified headache syndromes, R51 headache). Dementia was defined as an existing outpatient visitation record from January 1, 2006 to December 31, 2013. ICD-10 codes, AD (F00 dementia in Alzheimer’s disease, G30 Alzheimer’s disease) or VD (F01 vascular dementia) or unspecified dementia (F03 unspecified dementia, F02.8 dementia in other specified diseases classified elsewhere, G31.00 behavioral variant frontotemporal dementia, G31.82 dementia with Lewy bodies) were used. At the end of follow-up, the survival length was calculated until one of the following results appeared: (1) diagnosis of dementia; (2) death during follow-up period; (3) end of the year 2013.

### Statistical analyses

To compare the characteristics of the groups according to headache type, categorical data were provided as a percentage of the population using the Pearson chi-square test, and continuous data were provided as mean and standard deviation using the t-test. After adjusting potential confounding factors, the hazard ratios (HR) and 95% confidence intervals (95% CI) were calculated with the Cox proportional hazards regression model. Kaplan-Meier survival analysis was done to plot the survival probability throughout an 8-year follow-up period and the mean survival time for all-cause dementia according to the types of headaches, which includes the log survival function and log-rank test. Also, Kruskal-Wallis test with Bonferroni correction was used to compare the mean survival time for dementia according to headache type. Subgroup analysis was performed using sex, smoking status, alcohol intake, and CCI categories to identify relevant subgroups with potential relationships between headache and dementia. Statistical significance was set at a p-value <0.05. SAS 9.4 was utilized for all data collecting and statistical analysis (SAS Institute, Cary, NC, USA).

## Results

### Effect of headache on the incidence of dementia

Descriptive characteristics of the study population are shown in Table 1. A total of 470,652 (men: 53.2%, women 46.8%) participants were identified in the NHIS-HEAS cohort prior to the index date.

**Table 1:** Demographic and clinical characteristics of the study population according to headache type.

	Tension type headache	No tension type headache	P value	Migraine	No migraine	P value	Total
Number of subjects, n, (%)	34,124 (7.3)	436,528 (92.2)		29,999 (6.4)	440,653 (93.6)		470,652
Incidence rate for AD, (95% CI)^a^	67.6 (58.0–80.9)	39.5 (34.2–46.7)	<0.001	68.4 (18.6–82.1)	39.7 (34.4–46.9)	<0.001	41.5 (35.9–49.1)
Incidence rate for VD, (95% CI)^a^	23.6 (20.6–27.6)	14.8 (12.9–17.3)	<0.001	23.7 (20.6–27.8)	14.2 (12.4–16.6)	<0.001	15.4 (13.4–18.2)
Age, years, mean (SD)	57.2 (9.7)	55.3 (9.5)		57.1 (9.9)	55.3 (9.5)		55.5 (9.5)
Sex, %							
Male	35.2	54.7	<0.001	31.7	54.7	<0.001	53.2
Female	64.8	45.3		68.3	45.3		46.8
BMI, kg/m^2^, %							
< 25	64.4	65.4	<0.001	65.2	65.3	0.38	65.3
≥ 25	35.6	34.6		34.8	34.7		34.7
Household income, quartile, %							
1st (highest)	30.7	34.2	<0.001	30.1	34.2	<0.001	33.9
2^nd^	29.6	28.9		29.6	28.2		28.9
3^rd^	23.5	21.7		24.0	21.7		21.9
4th (lowest)	16.2	15.2		16.6	15.2		15.3
Smoking status, %							
Never	81.1	68.8	<0.001	82.5	68.8	<0.001	49.7
< 10 years	2.7	3.9		2.5	3.9		3.9
10–29 years	4.2	7.4		3.8	7.4		7.2
≥ 30 years	12.0	19.9		11.2	19.9		19.3
Alcohol consumption, per week, %							
Never	70.8	58.0	<0.001	72.5	58.0	<0.001	58.9
< 3 times	22.1	30.9		20.8	30.9		30.2
≥ 3 times	7.1	11.1		6.6	11.1		10.8
Physical exercise, per week, %							
Never	59.8	53.7	<0.001	60.8	53.7	<0.001	54.2
< 3 times	20.4	24.9		19.8	24.9		24.6
≥ 3 times	19.8	21.4		19.4	21.4		21.3
Blood pressure, mmHg, %							
SBP < 140 and DBP < 90	69.6	70.0	0.08	69.9	69.9	0.45	69.9
SBP ≥ 140 or DBP ≥ 90	30.4	30.0		30.1	30.1		30.1
Fasting serum glucose, mg/dL, %							
< 126	93.1	91.9	<0.001	93.4	91.9	<0.001	92.0
≥ 126	6.9	8.1		6.6	8.1		8.0
Total cholesterol, mg/dL, %							
< 200	51.0	53.5	<0.001	51.3	53.5	<0.001	53.3
≥ 200	49.0	46.5		48.7	46.5		46.7
Depression, n, (%)							
No	32,563 (95.4)	434,361 (99.5)	<0.001	28,827 (96.1)	438,097 (99.4)	<0.001	466,924 (99.2)
Yes	1,561 (4.6)	2,167 (0.5)		1,172 (3.9)	2,556 (0.6)		3,728 (0.8)
Sleep disorders, n, (%)							
No	32,736 (95.9)	434,468 (99.5)	<0.001	28,972 (96.6)	438,232 (99.5)	<0.001	467,204 (99.3)
Yes	1,388 (4.1)	2,060 (0.5)		1,027 (3.4)	2,421 (0.5)		3,448 (0.7)
Parkinson’s disease, n, (%)							
No	34,075 (99.9)	436,436 (100)	<0.001	29,972 (99.9)	440,539 (100)	<0.001	470,511 (100)
Yes	49 (0.1)	92 (0)		27 (0.1)	114 (0)		141 (0)
Head injury, n, (%)							
No	33,796 (99)	435,918 (99.9)	<0.001	29,773 (99.2)	439,941 (99.8)	<0.001	469,714 (99.8)
Yes	328 (1)	610 (0.1)		226 (0.8)	712 (0.2)		938 (0.2)
CCI, %							
0	6.3	14.9	<0.001	6.3	14.9	<0.001	14.3
1–2	33.4	42.8		33.4	42.7		42.2
≥ 3	60.3	42.2		60.2	42.4		43.5

Continuous variables are expressed as mean (SD), and categorical variables as %.

T-test for continuous variables and Chi-square test for categorical variables.

^a^per 10^4^ person-years.

Abbreviations: n, number of people; AD, alzheimer’s disease; VD, Vascular dementia; SD, standard deviation; CI, confidence intervals; SBP, systolic blood pressure; DBP, diastolic blood pressure; BMI, body mass index; CCI, Charlson comorbidity index.

The overall incidence rate for AD and VD were 41.5 and 15.4 per 10^4^ person-years, respectively. Patients diagnosed with TTH (AD p<0.001, VD p<0.001) and migraine (AD p<0.001, VD p<0.001) had a higher incidence rate. Regardless of the type of headache, headache patients had a higher proportion of women (p = <0.001) and higher total cholesterol (p<0.001), and CCI (p<0.001) values. In particular, headache patients had approximately 1.5 times more participants with 3 or more comorbidities (CCI≥3, p<0.001). Smoking (p<0.001) and alcohol intake (p<0.001), which reflect unhealthy lifestyles, were less evident in headache patients. Interestingly, conditions with known associations with dementia such as depression (p<0.001) and sleep disorders (p<0.001) were 4 to 10 times more frequent in headache patients.

**Fig 2:**
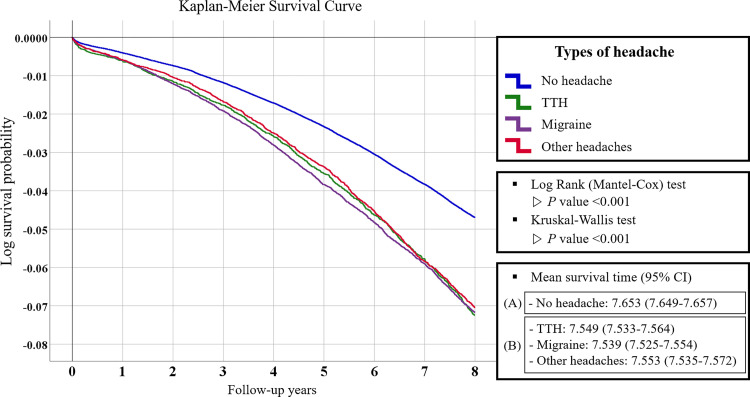
Kaplan–Meier survival curve for log survival probability of all-cause dementias according to headache type over 8 years follow up. Analysis of variance and Bonferroni’s post hoc analysis were performed to compare the difference in mean survival time between the ^a^no headache group and the ^b^headache group. X-axis = follow-up years, Y-axis = log survival probability. TTH = tension type headache.

For crude causal relationship of all cause dementias by type of headache, Kaplan–Meier survival curves with Log rank tests were performed (Fig 2). Survival time was significantly longer for no headache group (mean survival time, 7.653; 95% CI, 7.649–7.657) compared to the headache group (mean survival time, 7.547 years; 95% CI, 7.531–7.563) (P<0.001). This was true for all types of headaches analyzed (TTH mean survival time, 7.549; 95% CI, 7.533–7.564; migraine mean survival time, 7.539; 95% CI, 7.525–7.554, other headaches mean survival time, 7.553; 95% CI, 7.535–7.572). According to Kruskal-Wallis test and Bonferroni correction, there was no significant difference in average survival rates among headache types.

### Hazard ratios of dementias with headaches and subgroup analysis

We performed Cox proportional hazard regression analyzes to identify a multivariable association between headache and dementia after adjusting for age, sex, household income, smoking status, alcohol intake, physical activity, BMI, systolic blood pressure, fasting glucose, total cholesterol, CCI, and other known dementia-related comorbidities. As shown in Table 2, all headache types were associated with an elevated risk of all-cause dementia. TTH, migraine, and other primary headaches had similar HRs regardless of the type of dementia.

**Table 2:** Hazard ratios for dementias according to headache type.

	aHR (95% CI)			
	Alzheimer’s disease	Vascular dementia	Other dementia	All-cause dementia
Migraine				
Number of cases	1,539	548	867	2,164
cHR (95% CI)	**1.71 (1.62–1.80)**	**1.67 (1.53–1.82)**	**1.67 (1.56–1.79)**	**1.68 (1.61–1.76)**
aHR (95% CI)	**1.18 (1.12–1.24)**	**1.21 (1.10–1.32)**	**1.16 (1.08–1.25)**	**1.18 (1.13–1.24)**
Tension type headache				
Number of cases	1,739	602	992	2,435
cHR (95% CI)	**1.71 (1.62–1.79)**	**1.70 (1.48–1.76)**	**1.68 (1.58–1.80)**	**1.67 (1.60–1.74)**
aHR (95% CI)	**1.18 (1.13–1.25)**	**1.17 (1.07–1.27)**	**1.19 (1.11–1.27)**	**1.18 (1.13–1.24)**
Other primary headaches				
Number of cases	2,455	922	1,426	3,518
cHR (95% CI)	**1.66 (1.59–1.74)**	**1.73 (1.61–1.86)**	**1.67 (1.58–1.77)**	**1.67 (1.61–1.73)**
aHR (95% CI)	**1.17 (1.07–1.17)**	**1.22 (1.14–1.32)**	**1.14 (1.08–1.21)**	**1.15 (1.11–1.19)**
All-type Headache				
Number of cases	4,461	1,631	2,556	6,346
cHR (95% CI)	**1.71 (1.65–1.77)**	**1.73 (1.63–1.83)**	**1.70 (1.62–1.78)**	**1.70 (1.65–1.75)**
aHR (95% CI)	**1.15 (1.10–1.19)**	**1.23 (1.15–1.30)**	**1.16 (1.10–1.21)**	**1.16 (1.13–1.20)**

Adjusted hazard ratio calculated by Cox proportional hazards regression analysis after adjustments for age, sex, BMI, household income, smoking status, alcohol consumption, physical exercise, blood pressure, fasting serum glucose, total cholesterol, depression, sleep disorders, Parkinson’s disease, head injury, and Charlson Comorbidity Index. Bold indicates P < .05.

Abbreviations: cHR, crude hazard ratio; aHR; adjusted hazard ratio; CI, confidence intervals.

Sensitivity analysis results showed that dementia risk did not show a meaningful change from 1 to 5 years following the index date, except for participants diagnosed with dementia. After an incubation period of 5 years for dementia, the risk of dementia increased by 3% in TTH (aHR = 1.21, 95% CI = 1.14–1.28) and 1% in migraine patients (aHR = 1.19; 95% CI = 1.12–1.27). The direction of the results did not alter considerably when taking the development of dementia and the exclusion of relatively young participants during the incubation period into account.

The results from stratified analyses on the association of headache and dementia according to subgroups of sex, smoking status, alcohol consumption, and CCI are described in Table 3. Female patients with TTH and migraine had a higher risk of dementia (TTH, aHR = 1.19; 95% CI = 1.13–1.25; migraine, aHR = 1.19; 95% CI = 1.13–1.26) compared to male patients (TTH, aHR = 1.17; 95% CI = 1.08–1.28; migraine, aHR = 1.15; 95% CI = 1.05–1.26). Headache diagnosis in non-smokers was more sensitive to dementia than in smokers (TTH, aHR = 1.18; 95% CI = 1.13–1.24; migraine, aHR = 1.20; 95% CI = 1.14–1.26). The risk of dementias was higher in migraine patients with alcohol consumption, however this trend was not true in TTH patients. Among the participants without comorbidities (CCI = 0), migraine diagnosis did not significantly affect dementia risk, while the difference for dementia was significant according to TTH diagnosis (aHR = 1.22; 95% CI = 1.07–1.39).

**Table 3:** Subgroup analysis of associations between headaches and the risk of all cause dementia.

		Non-migraine	Migraine	Non-tension type headache	Tension type headache
Sex	Male				
	Number of cases	7,873	513	7,764	622
	aHR (95% CI)	1.00	**1.15 (1.05–1.26)**	1.00	**1.17 (1.08–1.28)**
	Female				
	Number of cases	11,403	1,651	11,241	1,813
	aHR (95% CI)	1.00	**1.19 (1.13–1.26)**	1.00	**1.19 (1.13–1.25)**
Smoking status	Non-smoker				
	Number of cases	14,939	1,867	14,732	2,074
	aHR (95% CI)	1.00	**1.20 (1.14–1.26)**	1.00	**1.18 (1.13–1.24)**
	Smoker (≤ 10 years)				
	Number of cases	414	33	409	38
	aHR (95% CI)	1.00	1.00 (0.70–1.44)	1.00	0.99 (0.70–1.39)
	Smoker (10≥ years, < 20 years)				
	Number of cases	514	30	497	47
	aHR (95% CI)	1.00	1.10 (0.76–1.60)	1.00	1.23 (0.90–1.68)
	Smoker (≥ 20 years)				
	Number of cases	3,409	234	3,367	276
	aHR (95% CI)	1.00	1.11 (0.97–1.27)	1.00	**1.18 (1.04–1.34)**
Alcohol consumption	Non-drinker				
	Number of cases	14,186	1,773	13,963	1,996
	aHR (95% CI)	1.00	**1.17 (1.12–1.23)**	1.00	**1.18 (1.13–1.24)**
	Per week, < 3 times				
	Number of cases	3,184	260	3,157	287
	aHR (95% CI)	1.00	**1.22 (1.07–1.39)**	1.00	**1.16 (1.03–1.31)**
	Per week, ≥ 3 times				
	Number of cases	1,906	131	1,885	152
	aHR (95% CI)	1.00	**1.22 (1.02–1.45)**	1.00	**1.22 (1.02–1.44)**
CCI	CCI = 0				
	Number of cases	11,241	1,813	5,302	854
	aHR (95% CI)	1.00	1.05 (0.91–1.22)	1.00	**1.22 (1.07–1.39)**
	CCI = 1 or 2				
	Number of cases	10,213	1,120	10,103	1,230
	aHR (95% CI)	1.00	**1.16 (1.10–1.24)**	1.00	**1.14 (1.07–1.21)**
	CCI ≥ 3				
	Number of cases	5,302	854	5,202	954
	aHR (95% CI)	1.00	**1.15 (1.07–1.24)**	1.00	**1.16 (1.08–1.25)**

Adjusted hazard ratio calculated by Cox proportional hazards regression analysis after adjustments for age, sex, BMI, household income, smoking status, alcohol consumption, physical exercise, blood pressure, fasting serum glucose, total cholesterol, depression, sleep disorders, Parkinson’s disease, head injury, and Charlson Comorbidity Index. Bold indicates P < .05.

Abbreviations: aHR; adjusted hazard ratio; CI, confidence intervals; CCI, Charlson comorbidity index.

## Discussion

The results of this study showed that headache diagnosis was associated with a significantly elevated risk of all types of dementias regardless of headache type even after adjusting for well-known confounders of dementia. This suggests a possible role of headache in the pathophysiology of dementia and need to consider active headache treatment to prevent dementia as an unfavorable comorbidity.

The incidence rates of 41.5 for AD and 15.4 for VD per 10^4^ person-years were similar to those found in a recent study based on 7 large cohorts in Europe and the United States [27]. Most researches on the association between headache and dementia have focused on the impact of migraine. The HR deduced in this study for migraine was 1.18 for all-cause dementia, 1.18 for AD, and 1.21 for VD. Such values are lower than those described in a previous study reporting HRs of 1.39 to 1.64 for all-cause dementia according to sex and educational levels [28]. Another study reported a HR for all-cause dementia of 1.44 in those with migraine [29]. A study that considered only AD as the outcome suggested a more consistent value with our study as an adjusted odds ratio of 1.13 for those with migraine [30]. A recent meta-analysis reported a relative risk of 1.34 for all-cause dementia and 2.49 for AD associated with migraine [31]. Such variations in HR values could be explained by differences in the factors considered for adjustment in analysis, patient definition, and age range of the study population, which suggests the need of a standardized data collection and analysis approach including migraine sub-diagnoses for deriving comparable results.

Unlike the results from the meta-analysis on migraine and dementia that did not find an association between migraine and VD [31] the risk was similarly increased for both VD (HR 1.21) and AD (HR 1.18) in the migraine population of our study. Another study also described that migraineurs had a significantly higher incidence of AD however, this was not true for VD [29]. Vascular problems leading to increased oxidative stress and structural brain changes identified in migraine are considered to contribute to the development of dementia [14–17]. In spite of the fact that it is reasonable to speculate an increased risk also for VD with migraine considering its vascular components [32] such heterogeneous results may reflect the complex interrelationship between cardiovascular disturbance and brain white matter abnormalities of both migraine [33] and dementia [34], and comorbidities including vascular problems may act as a mediating factor. This was reflected in our results showing migraine diagnosis not affecting dementia risk in participants without comorbidities. Also, it is common for AD and VD to coexist due to considerable overlap in the vascular pathology involved in AD [35]. So the impact of migraine on the development of VD should not be easily denied based on results that show otherwise.

Studies on the association between dementia and TTH as an independent entity are relatively sparse compared to those on migraine. As with previous literature persistently supporting an increased risk of dementia with migraine, TTH is also established as a risk factor of dementia. The risk for all types of dementia were significantly increased in our study with TTH and the HR for all-cause dementia was 1.18. A previous study based on a large cohort reported a similar HR of 1.15 after adjusting multiple confounding factors [36]. Another population based study reported a higher HR of 1.77 for all-cause dementia with TTH [34]. Unlike the results of our study showing all-type headaches with a higher HR for VD, TTH was associated with a higher HR for AD. This is in line with both previous studies showing a significantly increased risk only for nonvascular dementia with TTH [36, 37]. TTH is the most prevalent type of headache in the general population and also in those with dementia [38]. As with migraine, gray and white matter changes of the brain have been observed in TTH patients and this may act as the underlying mechanism that links TTH and dementia [39, 40]. The psychological burden and stress due to headache disorders could be another contributing factor to the increased risk of dementias as psychological stress induced immune response could facilitate the onset of neuro-inflammatory dementia [41, 42]. Stress is also associated with hypothalamic-pituitary-adrenal axis activation leading to increased levels of stress hormones that may cause neuronal atrophy [43]. As with dementias consisted of a wide spectrum of diseases that show overlapping features, migraine and TTH are also progressive conditions that share many clinical components and interactions in pathophysiology [44]. It is difficult to strictly separate both entities due to such commonalties and this may have caused the inconsistencies in results reporting on the relationship between dementia and headache as separate entities or as any headache as another meta-analysis did not find a significant association with any headache and AD while still showing an association with greater risk for all-cause dementia [13].

Subgroup analyses showed that female headache patients had a higher risk of dementia compared to males, which is consistent with previous results showing a higher rate of dementia in females with migraine [28–30], and TTH [36]. Unlike most relevant studies that lack important data on lifestyle, the results of our study showed that non-smokers with headaches were more sensitive to dementia than smokers. Unfavorable lifestyle itself is associated with increased dementia risk and headache may have a stronger influence in those with healthy lifestyles [45].

Despite the heterogeneity in diagnosis other primary headaches also increased the risk of dementia and this association was stronger with VD. Most previous studies did not report on the independent effect of other primary headache disorders and the results of this study showing an increased risk of all types of dementia with any type of headache underlines the need of general screening for cognitive decline and dementia in the headache population for early detection and timely management of both conditions. In interpreting the effect of headache on dementia risk, chronic migraine and chronic daily headache diagnoses should be approached with awareness. Since the diagnostic criteria of chronic migraine is under debate and clinical characteristics of migraine and TTH become less distinct with aging, the differential diagnosis headache subtypes may become questionable [46, 47].

There are several limitations of this study to consider in interpreting the results. First, the results of this study do not directly support a causal relationship between headache and dementia due to its retrospective nature. However, considering the long preclinical period of dementia, the timing of headache and dementia was separated by sensitivity analyses by verifying the initiation of follow-up by 1 year to 5 years after the index date to secure a temporal relationship and reduce the risk of underlying factors such as cerebrovascular mechanisms related to dementia affecting the risk of headache. Second, due to nature of the study based on diagnostic codes all possible confounding factors of both headache and dementia may not have been located and considered in analysis and this may have affected the results. Also, headache diagnosis itself could have been compromised due to the overlooking of change in headache features with aging. However, the finding of this study still hold important clinical implications since many well-known risk factors of dementia including age, sex, household income, lifestyle factors, physical activity, BMI, and various systemic conditions were adjusted when calculating HRs [48], and despite a higher prevalence of comorbidities with any headache, multivariate Cox analyses showed that headache was still an independent predictor for subsequent dementia risk. Also, the sub-analysis conducted in this study did not include headache intensity, duration, and frequency which may have resulted in the loss of detailed information related to the mechanism of headache and dementia, and further clinical studies are needed to verify such influences. Also, future studies involved in the development of artificial intelligence models based on big data to predict the development of dementia in headache patients would be able to overcome such limitations and provide a meaningful tool in enhancing awareness of chronic headaches and its treatment needs [49–51].

## Conclusions

The findings of this nationwide population-based study support the role of headache as a risk factor for dementia. The similar rate of dementia in individuals with both TTH and migraine, emphasizes the need for preventative measures regardless of headache type and more rigorous interventions in females and relatively healthy patients. Since headache, a debilitating condition in itself, is closely related to increased dementia risk which is known to cause higher individual and public costs the early diagnosis and timely treatment of headache is crucial. Future research should focus on further establishing pathological mechanisms and potential preemptive diagnostic measures related to dementias to be taken in headache populations.

## Supporting information

S1 Checklist STROBE statement—checklist of items that should be included in reports of observational studies. (DOCX)

S1 Table Sensitivity analysis according to incubation period on the association of tension type headache on dementia. (DOCX)

S2 Table Sensitivity analysis according to incubation period on the association of migraine on dementia. (DOCX)

## Abbreviations

aHRs: adjusted hazard ratios
CIs: confidence intervals
TTH: tension type headache
AD: Alzheimer’s disease
VD: vascular dementia
NHIS-HEALS: national health insurance service-national health screening cohort
CCI: Charlson comorbidity index
ICD: international classification of diseases
SBP: systolic blood pressure
DBP: diastolic blood pressure
BMI: body mass index
cHR: crude hazard ratio
