# Supplementary material for: Primary headaches increase the risk of dementias: An 8-year nationwide cohort study
Source: PLoS One. 2022 Aug 18;17(8):e0273220. doi: 10.1371/journal.pone.0273220 (PMC9387842; doi:10.1371/journal.pone.0273220)
Supplement: S1 Checklist — (DOCX) [file pone.0273220.s001.docx]

STROBE Statement—checklist of items that should be included in reports of observational studies

|  | Item No. | Recommendation | Page  No. | Relevant text from manuscript |
| --- | --- | --- | --- | --- |
| **Title and abstract** | 1 | (*a*) Indicate the study’s design with a commonly used term in the title or the abstract | 1 | “Primary headaches increase the risk of dementias: an 8-year nationwide cohort study” was described in the title. |
|  |  | (*b*) Provide in the abstract an informative and balanced summary of what was done and what was found | 3-4 | They were described in the Methods and Results in the abstract. |
| Introduction | | | |  |
| Background/rationale | 2 | Explain the scientific background and rationale for the investigation being reported | 5 | They were described using the citied references in the Background. |
| Objectives | 3 | State specific objectives, including any prespecified hypotheses | 6 | “Therefore, this longitudinal population-based cohort study based on large datasets applying advanced analytic strategies aimed to explore the possible causal relationship between different types of headaches and dementias to define the role of headache treatment for the prevention of incident dementia.” were described in the last paragraph of the Background. |
| Methods | | | |  |
| Study design | 4 | Present key elements of study design early in the paper |  | “The National Health Insurance Service-National Health Screening Cohort (NHIS-HEALS) data were used in this retrospective cohort study.” Were described in the beginning of the Methods |
| Setting | 5 | Describe the setting, locations, and relevant dates, including periods of recruitment, exposure, follow-up, and data collection |  | “Study population, definition of clinical outcomes, covariates, and data analyses” were described in the Methods in detail. |
| Participants | 6 | (*a*) *Cohort study*—Give the eligibility criteria, and the sources and methods of selection of participants. Describe methods of follow-up  *Case-control study*—Give the eligibility criteria, and the sources and methods of case ascertainment and control selection. Give the rationale for the choice of cases and controls  *Cross-sectional study*—Give the eligibility criteria, and the sources and methods of selection of participants |  | “A subject sampling and the used data” were described in the beginning of the Methods. |
|  |  | (*b*) *Cohort study*—For matched studies, give matching criteria and number of exposed and unexposed  *Case-control study*—For matched studies, give matching criteria and the number of controls per case | N/a | N/a |
| Variables | 7 | Clearly define all outcomes, exposures, predictors, potential confounders, and effect modifiers. Give diagnostic criteria, if applicable |  | “Migraine, tension type headache, Alzheimer’s disease, vascular dementia as the important variables and covariates” were described in the Methods in detail. |
| Data sources/ measurement | 8* | For each variable of interest, give sources of data and details of methods of assessment (measurement). Describe comparability of assessment methods if there is more than one group |  | “The details of assessment of main exposures, outcomes and other variables” were described in the section of “Definition of clinical outcomes and Covariates” in the Methods. |
| Bias | 9 | Describe any efforts to address potential sources of bias |  | They were described in the discussion. |
| Study size | 10 | Explain how the study size was arrived at | 7 | “Of the 509,900 participants who maintained insurance eligibility in 2005 (index date: January 1, 2006), 2,938 deaths occurred before the index date and 36,310 participants without screening data were excluded. Then, cases of dementia diagnosed from 2002 to 2005 were also excluded as shown in fig. 1.” were described in methods and Figure 1. |

Continued on next page

| Quantitative variables | 11 | Explain how quantitative variables were handled in the analyses. If applicable, describe which groupings were chosen and why |  | They were described in the Methods, and presented mainly in the Data analysis. |
| --- | --- | --- | --- | --- |
| Statistical methods | 12 | (*a*) Describe all statistical methods, including those used to control for confounding |  | They were described in the Statistical analysis. |
|  |  | (*b*) Describe any methods used to examine subgroups and interactions |  | There were no significant interactions. |
|  |  | (*c*) Explain how missing data were addressed |  | “Of the 509,900 participants who maintained insurance eligibility in 2005 (index date: January 1, 2006), 2,938 deaths occurred before the index date and 36,310 participants without screening data were excluded. Then, cases of dementia diagnosed from 2002 to 2005 were also excluded as shown in fig. 1.” were described in methods and Figure 1. |
|  |  | (*d*) *Cohort study*—If applicable, explain how loss to follow-up was addressed  *Case-control study*—If applicable, explain how matching of cases and controls was addressed  *Cross-sectional study*—If applicable, describe analytical methods taking account of sampling strategy |  |  |
|  |  | (*e*) Describe any sensitivity analyses |  | “After excluding participants diagnosed with dementia up to 5 years after the index date, a sensitivity analysis on the association of headache on dementia was performed..” were described in methods. |
| Results | | | | |
| Participants | 13* | (a) Report numbers of individuals at each stage of study—eg numbers potentially eligible, examined for eligibility, confirmed eligible, included in the study, completing follow-up, and analysed |  | “A total of subjects and the distribution” were describe in the Results and Table 1. |
|  |  | (b) Give reasons for non-participation at each stage | N/a | N/a |
|  |  | (c) Consider use of a flow diagram |  | A flowchart for study population selection was illustrated in Figure 1. |
| Descriptive data | 14* | (a) Give characteristics of study participants (eg demographic, clinical, social) and information on exposures and potential confounders |  | They were described in the Table 1. |
|  |  | (b) Indicate number of participants with missing data for each variable of interest |  | They were described in the Table 1. |
|  |  | (c) *Cohort study*—Summarise follow-up time (eg, average and total amount) |  | Follow-up time was described in abstract and figure 2. |
| Outcome data | 15* | *Cohort study*—Report numbers of outcome events or summary measures over time |  | They were described in the Table 1. |
|  |  | *Case-control study—*Report numbers in each exposure category, or summary measures of exposure | N/a | N/a |
|  |  | *Cross-sectional study—*Report numbers of outcome events or summary measures | N/a | N/a |
| Main results | 16 | (*a*) Give unadjusted estimates and, if applicable, confounder-adjusted estimates and their precision (eg, 95% confidence interval). Make clear which confounders were adjusted for and why they were included |  | They were described in the Table 2 ,3 and the descriptions were described in the Results. |
|  |  | (*b*) Report category boundaries when continuous variables were categorized |  | They were described in the Methods. |
|  |  | (*c*) If relevant, consider translating estimates of relative risk into absolute risk for a meaningful time period | N/a | N/a |

Continued on next page

| Other analyses | 17 | Report other analyses done—eg analyses of subgroups and interactions, and sensitivity analyses |  | subgroups and sensitivity analyses were described in Results |
| --- | --- | --- | --- | --- |
| Discussion | | | | |
| Key results | 18 | Summarise key results with reference to study objectives |  | “The results of this study showed that headache diagnosis was associated with a significantly elevated risk of all types of dementias regardless of headache type even after adjusting for well-known confounders of dementia.” were described in Discussion |
| Limitations | 19 | Discuss limitations of the study, taking into account sources of potential bias or imprecision. Discuss both direction and magnitude of any potential bias |  | The limitations and any potential bias were described in the last paragraph of Discussion. |
| Interpretation | 20 | Give a cautious overall interpretation of results considering objectives, limitations, multiplicity of analyses, results from similar studies, and other relevant evidence |  | They were described in Discussion. |
| Generalisability | 21 | Discuss the generalisability (external validity) of the study results |  | They were described in Discussion. |
| Other information | |  | | |
| Funding | 22 | Give the source of funding and the role of the funders for the present study and, if applicable, for the original study on which the present article is based |  | They were described in Acknowledgements. |

*Give information separately for cases and controls in case-control studies and, if applicable, for exposed and unexposed groups in cohort and cross-sectional studies.

**Note:** An Explanation and Elaboration article discusses each checklist item and gives methodological background and published examples of transparent reporting. The STROBE checklist is best used in conjunction with this article (freely available on the Web sites of PLoS Medicine at http://www.plosmedicine.org/, Annals of Internal Medicine at http://www.annals.org/, and Epidemiology at http://www.epidem.com/). Information on the STROBE Initiative is available at www.strobe-statement.org.
