# Supplementary material for: Primary headaches increase the risk of dementias: An 8-year nationwide cohort study
Source: PLoS One. 2022 Aug 18;17(8):e0273220. doi: 10.1371/journal.pone.0273220 (PMC9387842; doi:10.1371/journal.pone.0273220)
Supplement: S1 Table — (DOCX) [file pone.0273220.s002.docx]

**Table S1.** Sensitivity analysis according to incubation period on the association of tension type headache on dementia

|  |  | | Dementia incubation period, years | | | | |
| --- | --- | --- | --- | --- | --- | --- | --- |
|  | Non-tension type headache^a^ | 1 | | 2 | 3 | 4 | 5 |
| Overall dementia |  |  | |  |  |  |  |
| Events | 19,087 | 2,317 | | 2,143 | 1.909 | 1,603 | 1,273 |
| aHR (95% CI) | 1.00 (Ref.) | **1.17  (1.12-1.23)** | | **1.18  (1.13-1.24)** | **1.19  (1.13-1.25)** | **1.19  (1.13-1.26)** | **1.21  (1.14-1.28)** |
| Alzheimer’s disease |  |  | |  |  |  |  |
| Events | 13,229 | 1,673 | | 1,563 | 1,417 | 1,212 | 1,003 |
| aHR (95% CI) | 1.00 (Ref.) | **1.19  (1.13-1.25)** | | **1.19  (1.13-1.26)** | **1.20  (1.13-1.27)** | **1.20  (1.13-1.28)** | **1.23  (1.15-1.31)** |
| Vascular dementia |  |  | |  |  |  |  |
| Events | 4,990 | 590 | | 549 | 473 | 395 | 296 |
| aHR (95% CI) | 1.00 (Ref.) | **1.15  (1.05-1.25)** | | **1.16  (1.06-1.27)** | **1.16  (1.05-1.28)** | **1.20  (1.08-1.34)** | **1.17  (1.04-1.33)** |
| Other dementia |  |  | |  |  |  |  |
| Events | 7,586 | 950 | | 876 | 789 | 675 | 538 |
| aHR (95% CI) | 1.00 (Ref.) | **1.16  (1.08-1.25)** | | **1.16  (1.08-1.25)** | **1.18  (1.09-1.27)** | **1.17  (1.08-1.27)** | **1.19  (1.08-1.30)** |

^a^Participants without tension type headache before the index date

Abbreviations: aHR; adjusted hazard ratio; CI, confidence intervals
