# Supplementary material for: Primary headaches increase the risk of dementias: An 8-year nationwide cohort study
Source: PLoS One. 2022 Aug 18;17(8):e0273220. doi: 10.1371/journal.pone.0273220 (PMC9387842; doi:10.1371/journal.pone.0273220)
Supplement: S2 Table — (DOCX) [file pone.0273220.s003.docx]

**Table S2.** Sensitivity analysis according to incubation period on the association of migraine on dementia

|  |  | | Dementia incubation period, years | | | | |
| --- | --- | --- | --- | --- | --- | --- | --- |
|  | Non-migraine^a^ | 1 | | 2 | 3 | 4 | 5 |
| Overall dementia |  |  | |  |  |  |  |
| Events | 19,348 | 2,080 | | 1,912 | 1,720 | 1,441 | 1,133 |
| aHR (95% CI) | 1.00 (Ref.) | **1.17  (1.12-1.23)** | | **1.17  (1.12-1.23)** | **1.19  (1.13-1.26)** | **1.19  (1.13-1.26)** | **1.19  (1.12-1.27)** |
| Alzheimer’s disease |  |  | |  |  |  |  |
| Events | 13,191 | 1,478 | | 1,380 | 1,260 | 1,079 | 882 |
| aHR (95% CI) | 1.00 (Ref.) | **1.17  (1.11-1.23)** | | **1.17  (1.11-1.24)** | **1.19  (1.12-1.26)** | **1.19 (1.12-1.27)** | **1.20  (1.12-1.28)** |
| Vascular dementia |  |  | |  |  |  |  |
| Events | 5,032 | 553 | | 518 | 448 | 336 | 263 |
| aHR (95% CI) | 1.00 (Ref.) | **1.20  (1.10-1.32)** | | **1.22  (1.11-1.34)** | **1.22  (1.11-1.34)** | **1.23  (1.11-1.35)** | **1.20  (1.08-1.33)** |
| Other dementia |  |  | |  |  |  |  |
| Events | 7,814 | 832 | | 761 | 687 | 586 | 459 |
| aHR (95% CI) | 1.00 (Ref.) | **1.13  (1.05-1.21)** | | **1.12  (1.03-1.20)** | **1.13  (1.05-1.23)** | **1.12  (1.03-1.23)** | **1.12  (1.01-1.23)** |

^a^Participants without migraine before the index date

Abbreviations: aHR; adjusted hazard ratio; CI, confidence intervals
